# Development and validation of a nomogram model for predicting chronic kidney disease after liver transplantation: a multi-center retrospective study

**DOI:** 10.1038/s41598-023-38626-4

**Published:** 2023-07-14

**Authors:** Zenglei He, Yimou Lin, Siyi Dong, Qinghong Ke, Shusen Zheng, Qi Ling

**Affiliations:** 1 Department of Hepatobiliary and Pancreatic Surgery, The First Affiliated Hospital, Zhejiang University School of Medicine, Hangzhou, 310003 China; 2 China Liver Transplant Registry, Hangzhou, 310003 China

**Keywords:** Liver, Kidney, Risk factors

## Abstract

Chronic kidney disease (CKD) is a frequent complication after liver transplantation (LT) and associated with poor prognosis. In this study, we retrospectively analyzed 515 adult patients who underwent LT in our center. They were randomly divided into a training set (n = 360) and an internal test set (n = 155). Another 118 recipients in other centers served as external validation set. Univariate and multivariate COX regression analysis were used to determine risk factors. A nomogram model was developed to predict post-LT CKD. The incidence of post-LT CKD in our center was 16.9% (87/515) during a median follow-up time of 22.73 months. The overall survival of recipients with severe CKD (stage IV and V) were significantly lower than those with non or mild CKD (stage III) (*p* = 0.0015). A nomogram model was established based on recipient’s age, anhepatic phase, estimated glomerular filtration rate and triglyceride levels at 30 days after LT. The calibration curves for post-LT CKD prediction in the nomogram were consistent with the actual observation in both the internal and external validation set. In conclusion, severe post-LT CKD resulted in a significantly reduced survival in liver recipient. The newly established nomogram model had good predictive ability for post-LT CKD.

## Introduction

Liver transplantation (LT) is a life-saving treatment for patients with end-stage liver diseases. With the development of LT surgery technology and postoperative management, the 10-year survival rate after LT is nearly 89%^1^. However, the postoperative complications could strongly impair graft function and reduce graft survival. Chronic kidney disease (CKD) is one of the frequent long-term complications in liver recipients. According to the previous reports, the incidence of post-LT CKD ranged from 11.7% to 54%^2–10^. Based on data of 1771 liver recipients in Taiwan National Health Insurance Research Database, Wang et al.^7^ revealed that 323 (18.2%) patients required renal replacement therapy after LT and had a higher mortality.

The etiologies of post-LT CKD are multifactorial (e.g., perioperative kidney injury, recipient factors, the use of calcineurin-inhibitor) and the risk factors varied from each other in different studies^4^, making it difficult to accurately identify recipients who were at high risk of developing post-LT CKD. Over the past decade, models have been established and tried to serve as tools to predict the development of post-LT CKD. Giusto et al.^11^ analyzed data from 179 patients and found that arterial hypertension, severe infection and estimated glomerular filtration rate (eGFR) after LT were risk factors for the development of post-LT CKD. A predictive model was established based on these factors and showed a C-index of 0.91, indicating good predictive ability. Levitsky et al.^12^ proposed another predictive model by integrating one clinical parameter (hepatitis C virus as the major indication for LT) and two renal injury proteins (serum CD 40 antigen and β-2 microglobulin prior to LT), showing an area under the curve (AUC) of 0.814 in the training cohort (n = 60) and 0.801 in the validation cohort (n = 50). Nevertheless, the detection of these individual biomarkers seems to be difficult and increase the time cost as well, limiting their application in clinic. Furthermore, the sample sizes of the two models are relatively small, suggesting that larger cohorts should be considered to validate their efficacy. In a large cohort study with 43,514 adult liver recipients, Sharma et al.^13^ established a renal risk index, which contained 14 recipient factors, to predict end-stage renal disease (ESRD) after LT. However, the model could not identify patients with CKD stage III or IV who may deteriorate to CKD stage V without being well managed. In addition, it involved too many variables, which might increase the difficulty of clinical application. Therefore, a simple and effective predictive model is needed to identify patients at high risk of post-LT CKD.

Nomogram is a simple visual graph of statistical prediction model with an easy-to-use graphical interface. Importantly, it could generate personalized predictions, thereby, widely used in risk stratification and personally providing approach to disease management. In our study, we aimed to develop a nomogram predictive model for CKD following donation after circulatory death (DCD) LT.

## Methods

### Patient characteristics

All adult patients (> 18 years old) undergoing DCD LT between January 1, 2015 and December 31, 2018 at The First Affiliated Hospital, Zhejiang University School of Medicine were enrolled in this retrospective study. At first, 619 LT recipients were included, 10 with age of < 18 years and 94 who died within 3 months after LT were excluded. Then 515 patients were included and randomly divided into a training set (n = 360) and an internal test set (n = 155) according to ratio of 0.7 to 0.3^14,15^. An external validation set (n = 118) was obtained from China Liver Transplant Registry (CLTR) database and included adult patients who underwent primary LT from July 1, 2017 to December 31, 2020 in other centers with sufficient data. The study protocol was approved by the clinical ethics review board of hospital and CLTR. All LTs were performed with organs from voluntary donations made by deceased donors, not from executed prisoners. Each organ donation or transplant followed the guidelines of the Organ Transplant Committee of China. The informed consent was obtained from each participant.

### Data collection

Data related to demographic or perioperative variables were collected in accordance with the procedure described in previous studies^16^. The following perioperative clinical variables were recorded for the study: patient demographics, medical history, medication history, baseline laboratory findings, medical information about the donor and graft liver, operation time, intraoperative fluid and colloid administration, and intraoperative transfusion amount. The pre-transplant data were collected within 24 h before LT. The post-LT data were recorded during the follow up.

### Definition

The primary outcome variable was postoperative CKD defined according to Kidney Disease: Improving Global Outcomes (KDIGO) 2012 CKD Guideline. We used the following criteria: eGFR < 60 mL/min/1.73 m^2^ ≥ 3 months, with or without renal injury^17–19^. The eGFR calculated using the serum creatinine value and modified diet in renal disease (MDRD) equation^20^.

### Statistical analysis

R software version 4.1.0 (http://www.r-project.org/) were used for statistical analysis of data in this study. Quantitative variables were expressed as mean ± SD or median and quartiles. Categorical variables such as sex were presented as values and percentages. Student's t test or the Wilcoxon's rank-sum test was used to compare quantitative variables. The Chi-square test was used to compare categorical variables. The Kaplan–Meier method and log-rank test were used for survival analysis. Multivariate COX regression with LR forward elimination analysis was used to determine independent risk factors for post-LT CKD. We calculated the AUCs of the models and compared the AUCs using De Long’s method^21^. A nomogram was built and verified in the training set and two test sets respectively.

In this study, the ‘SurvMiner’ and ‘Survival’ packages in R language were used to conduct Cox regression analysis and establish a nomogram prediction model for post-LT CKD. The ‘tidyverse’, ‘SurvivalRoc’, ‘pROC’, ‘ROCR’ packages were used for AUC analysis. The ‘stdca.R’ source code (http://ame.pub/AME20210218) was used for DCA analysis of the models. P value < 0.05 was considered statistically significant.

### Ethical approval

This study was approved by the clinical ethics review board of The First Affiliated Hospital, Zhejiang University School of Medicine and CLTR.

### Informed consent

Informed consent was obtained from all patients to allow the use of clinical data for investigation.

## Results

### Basic characteristics results

A total of 633 adult DCD LT recipients were enrolled in this study. There were 523 males and 110 females. Median age at LT was 51.0 years (quartile: 42.0–57.0 years). The median follow-up time was 22.83 months (quartile: 13.62–39.67 months). The average time of CKD diagnosis after LT was 19.61 months (quartile: 11.25–37.34 months). Demographic and clinical characteristics were shown in Table 1. Other complications, such as biliary complications, acute rejection and early allograft dysfunction, showed no significances between our center and external validation set.

**Table 1:** Patient characteristics.

	Overall	Our center		Validation set	*p* value*
		Training set	Test set		
	n = 633	n = 360	n = 155	n = 118	
Donors					
Male (%)	532 (84.0)	302 (83.9)	127 (81.9)	103 (87.3)	0.485
Age (year)	45 (34, 53)	44 (34, 52)	43 (30, 51)	49 (40, 55)	**0.001**
BMI (kg/m^2^)	22.49 (20.76, 24.22)	22.70 (20.96, 24.22)	22.49 (21.16, 24.22)	22.49 (19.86, 24.22)	0.303
Graft Weight (g)	1375 (1195, 1577)	1380 (1194, 1579)	1364 (1195, 1524)	1447 (1200, 1642)	0.254
Warm ischemia (min)	8 (2, 13)	10 (4, 14)	9 (3, 13)	3 (0, 4)	** < 0.001**
Cold ischemia (hour)	8.50 (6.78, 10.93)	8.90 (6.82, 11.63)	9.08 (6.62, 11.43)	8.00 (7.00, 9.00)	** < 0.001**
Anhepatic phase (min)	68 (58, 80)	69 (60, 80)	69 (61, 85)	55 (43, 74)	** < 0.001**
Operation time (hour)	5.10 (4.50, 5.92)	5.11 (4.56, 5.92)	5.17 (4.57, 5.87)	5.00 (4.26, 5.99)	0.057
Recipients					
Male (%)	523 (82.6)	296 (82.2)	127 (81.9)	100 (84.7)	0.794
Age (year)	51 (42, 57)	51 (42, 58)	51 (43, 56)	50 (43, 56)	0.801
BMI (kg/m^2^)	22.68 (20.72, 24.80)	22.67 (20.59, 24.71)	22.58 (20.55, 24.96)	22.84 (20.87, 24.59)	0.618
MELD score	24 (13, 32)	22 (13, 31)	20 (11, 30)	32 (25, 39)	** < 0.001**
Child-Pugh score	10 (8, 11)	10 (7, 11)	9 (7, 11)	11 (10, 11)	** < 0.001**
History of hypertension (%)	87 (13.7)	52 (14.4)	20 (12.9)	15 (12.7)	0.841
History of diabetes (%)	93 (14.7)	58 (16.1)	20 (12.9)	15 (12.7)	0.511
History of hyperlipidemia (%)	14 (2.2)	8 (2.2)	3 (1.9)	3 (2.5)	0.944
Follow up duration (month)	22.83 (13.62, 39.67)	24.03 (13.91, 38.48)	21.02 (14.39, 37.03)	25.68 (13.11, 45.38)	0.498
Average time of CKD diagnosis (month)	19.61 (11.25, 37.34)	19.27 (9.63, 35.96)	20.66 (11.93, 36.35)	18.52 (12.36, 45.02)	0.119
CKD Stage (%)					0.224
non-CKD	534 (84.4)	300 (83.3)	128 (82.6)	106 (89.8)	
III	81 (12.8)	51 (14.2)	23 (14.8)	7 (5.9)	
IV	9 (1.4)	4 (1.1)	3 (1.9)	2 (1.7)	
V	9 (1.4)	5 (1.4)	1 (0.6)	3 (2.5)	
Other complications n (%)					
Biliary complication	113 (17.9)	69 (19.2)	25 (16.1)	19 (16.1)	0.611
Acute rejection	87 (13.7)	50 (13.9)	19 (12.3)	18 (15.3)	0.770
EAD	124 (19.6)	67 (18.6)	29 (18.7)	3 (2.5)	0.454
Laboratory results at 30 days after liver transplantation					
eGFR (mL/min/1.73 m^2^)	106.80 (85.89, 130.16)	104.66 (81.08, 130.37)	106.23 (88.33, 132.24)	111.60 (98.12, 126.74)	0.242
Albumin (g/L)	44.0 (40.00, 47.00)	45.00 (42.00, 48.00)	44.00 (42.00, 47.50)	37.85 (35.50, 41.90)	** < 0.001**
Triglyceride (mmol/L)	1.77 (1.32, 2.35)	1.83 (1.40, 2.44)	1.80 (1.44, 2.42)	1.49 (1.08, 1.95)	** < 0.001**
Fasting blood-glucose (mmol/L)	6.13 (5.34, 7.82)	6.36 (5.52, 8.35)	6.06 (5.47, 7.56)	5.42 (4.77, 7.06)	** < 0.001**
Creatinine (μmol/L)	72.00 (57.00, 88.00)	75.00 (61.00, 92.00)	74.00 (60.00, 87.00)	55.50 (43.25, 75.00)	** < 0.001**
Hemoglobin (g/L)	103 (89, 115)	105 (93, 116)	105 (93, 117)	88 (77, 103)	** < 0.001**

Abbreviations: *BMI* Body mass index; *CKD* Chronic kidney disease; *EAD* Early allograft dysfunction; *eGFR* Estimated glomerular filtration rate; *MELD* Model for end-stage liver disease.

Significant values are in bold.

*p value was calculated by the comparison between our center (n = 515) and validation set (n = 118).

### Incidence and prognosis of post-LT CKD in our center

**Figure 1:**
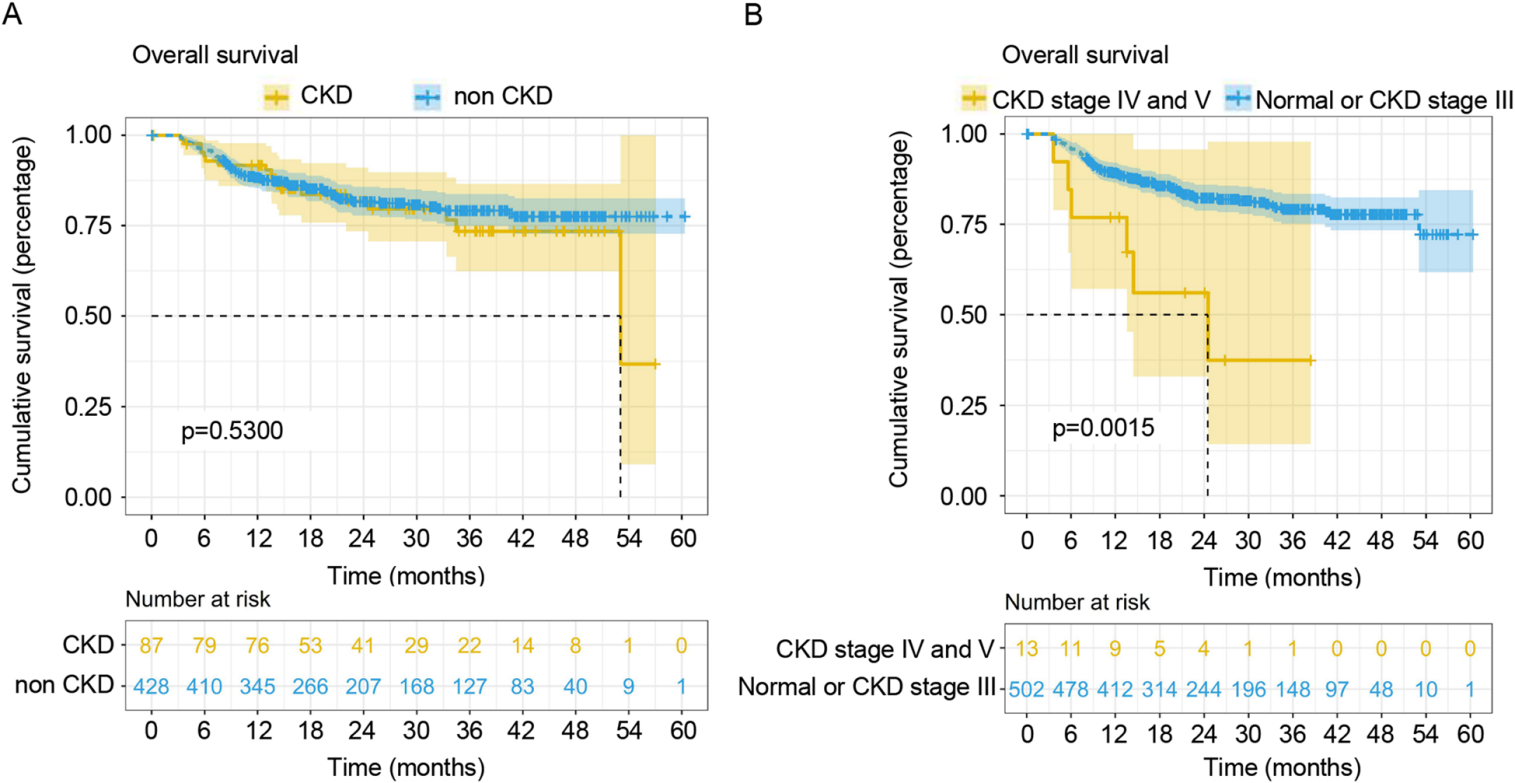
Cumulative patient survival after liver transplantation. (**A**) Comparison of cumulative patient survival between the CKD group and the non-CKD group; log-rank test, *p* = 0.5300. (**B**) Comparison of cumulative patient survival between the severe CKD stage group (CKD stage IV and CKD stage V) and the mild CKD stage (CKD stage III) or non-CKD group; log-rank test, *p* = 0.0015.

Out of 633 cases, 99 developed CKD. Among these 99 recipients, 18 deteriorated to CKD IV or CKD V and finally only 9 developed ESRD. In our center, the incidence of post-LT CKD was 16.9% (87/515; CKD stage III 14.4% n = 74; CKD IV 1.4% n = 7; CKD stage V 1.2% n = 6). The 1-, 2- and 3-year cumulative survival rates in the CKD group were 91.7%, 81.8% and 73.5% and the corresponding survival rates in the non-CKD group were 88.4%, 81.7% and 79.2% respectively. There was no statistically significant difference in survival between the two groups (*p* = 0.53) (Fig. 1A). However, the survival rates of severe CKD recipients (CKD stage IV and V) after LT decreased significantly. The cumulative survival rates at 1-, 2-, and 3-year were 76.9%, 56.1%, and 37.4% and the corresponding survival rates for non-CKD or CKD stage III recipients were 89.4%, 82.5%, and 79.3% respectively (p = 0.0015) (Fig. 1B).

### Risk factors of post-LT CKD

Due to the fewer cases of CKD IV, CKD V and ESRD, it was difficult to instruct a predictive model. Herein, we focused on post-LT CKD in all stages. In the training set, Cox regression univariate analysis showed that 23 variables were associated with post-LT CKD (p < 0.05) (Table 2). To predict the occurrence of post-LT CKD before LT, we firstly determined the independent preoperative variables using multivariable COX regression analysis with forward LR method. The results showed that recipient’s age (HR 1.04, 95%CI 1.01–1.06), body mass index (BMI) (HR 1.10, 95%CI 1.01–1.18), history of hepatorenal syndrome (HR 3.91, 95%CI 1.94–7.90) and anhepatic phase (HR 1.02, 95%CI 1.01–1.03) were independent risk factors for post-LT CKD in training set.

**Table 2:** Univariate and multivariate COX regression analysis.

Variables	Univariate COX regression analysis			Multivariate COX regression analysis		
	HR	95%CI	*p*	HR	95%CI	*p* value
Pre-operative and intra-operative variables						
Creatinine (μmol/L)	1.004	1.002–1.007	< 0.001			
Anhepatic phase (min)	1.018	1.008–1.029	0.001	1.020	1.010–1.031	< 0.001
Hepatorenal syndrome	0.352	0.178–0.695	0.003			
MELD	1.033	1.009–1.057	0.008			
Age (year)	1.033	1.007–1.060	0.012	1.027	1.001–1.053	0.039
Prothrombin time	1.460	1.068–1.994	0.017			
BMI	1.093	1.016–1.175	0.018			
History of dialysis	0.332	0.132–0.835	0.019			
Use of Entecavir	1.915	1.080–3.397	0.026			
History of cerebrovascular disease	0.364	0.145–0.912	0.031			
Use of Telbivudine	0.593	0.355–0.991	0.046			
Post-operation variables						
Length of stay in the care unit (days)	1.002	1.001–1.003	0.001			
Laboratory findings at 30-day after LT						
Cystatin C (mg/L)	1.922	1.625–2.273	< 0.001			
Urea nitrogen (mmol/L)	1.078	1.056–1.100	< 0.001			
Creatinine (μmol/L)	1.005	1.003–1.006	< 0.001			
eGFR (mL/min/1.73m^2^)	0.978	0.971–0.984	< 0.001	0.979	0.972–0.986	< 0.001
Uric acid (μmol/L)	1.003	1.002–1.005	< 0.001			
Triglyceride (mmol/L)	1.358	1.189–1.551	< 0.001	1.506	1.234–1.838	< 0.001
Fasting blood glucose (mmol/L)	1.106	1.055–1.160	< 0.001			
Hemoglobin (g/L)	0.970	0.955–0.985	< 0.001			
Albumin (g/L)	0.931	0.881–0.985	0.012			
Low density lipoprotein cholesterol (mmol/L)	0.641	0.446–0.919	0.016			

Abbreviations: *BMI* Body mass index; *eGFR* Estimated glomerular filtration rate; *HR* Hazard ratio; *MELD* Model for end-stage liver disease; *PT* Prothrombin time.

Because CKD is a chronic disease happened after 3 months postoperatively, we assumed that early postoperative variables might better predict the disease. Therefore, we next entered parameters at 30 days after LT into the multivariable COX regression model. The results showed that recipient’s age (HR 1.03, 95%CI 1.00–1.05), anhepatic phase (HR 1.02, 95%CI 1.01–1.03), eGFR at 30 days after LT (HR 0.98, 95%CI 0.97–0.99) and TG levels at 30 days after LT (HR 1.51 95%CI 1.23–1.83) were independent risk factors for post-LT CKD in training set (Table 2).

### Predictive models of post-LT CKD

**Figure 2:**
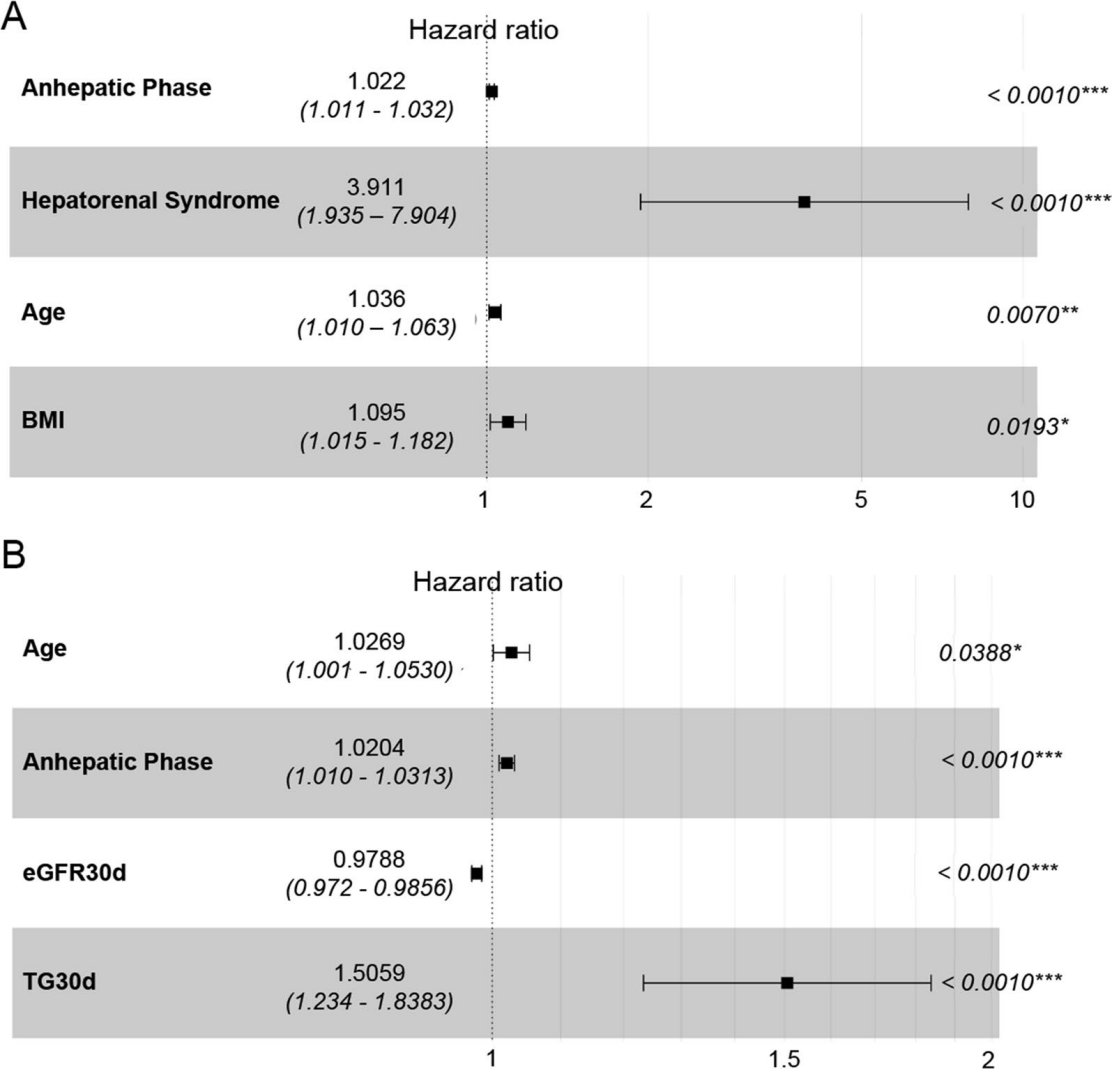
Multivariate COX regression analysis in the training set. (**A**) The four risk factors (Age, BMI, hepatorenal syndrome and anhepatic phase) included in model 1. (**B**) The four risk factors (Age, anhepatic phase, eGFR at 30 days after LT and TG levels at 30 days after LT) included in model 2. Abbreviations: *BMI* Body mass index; *eGFR30d* Estimated glomerular filtration rate at 30 days after LT (mL/min/1.73 m^2^); *TG30d* Triglyceride levels at 30 days after LT (mmol/L).

We established a preoperative prediction model (model 1) according to recipient’s age, BMI, history of hepatorenal syndrome and anhepatic phase (Fig. 2A). The AUCs of model 1 in training set, internal test set and external validation set were 0.6988 (95%CI 0.6233–0.7743), 0.6126 (95%CI 0.504–0.7291) and 0.6187 (95%CI 0.5101–0.7475) respectively.

**Figure 3:**
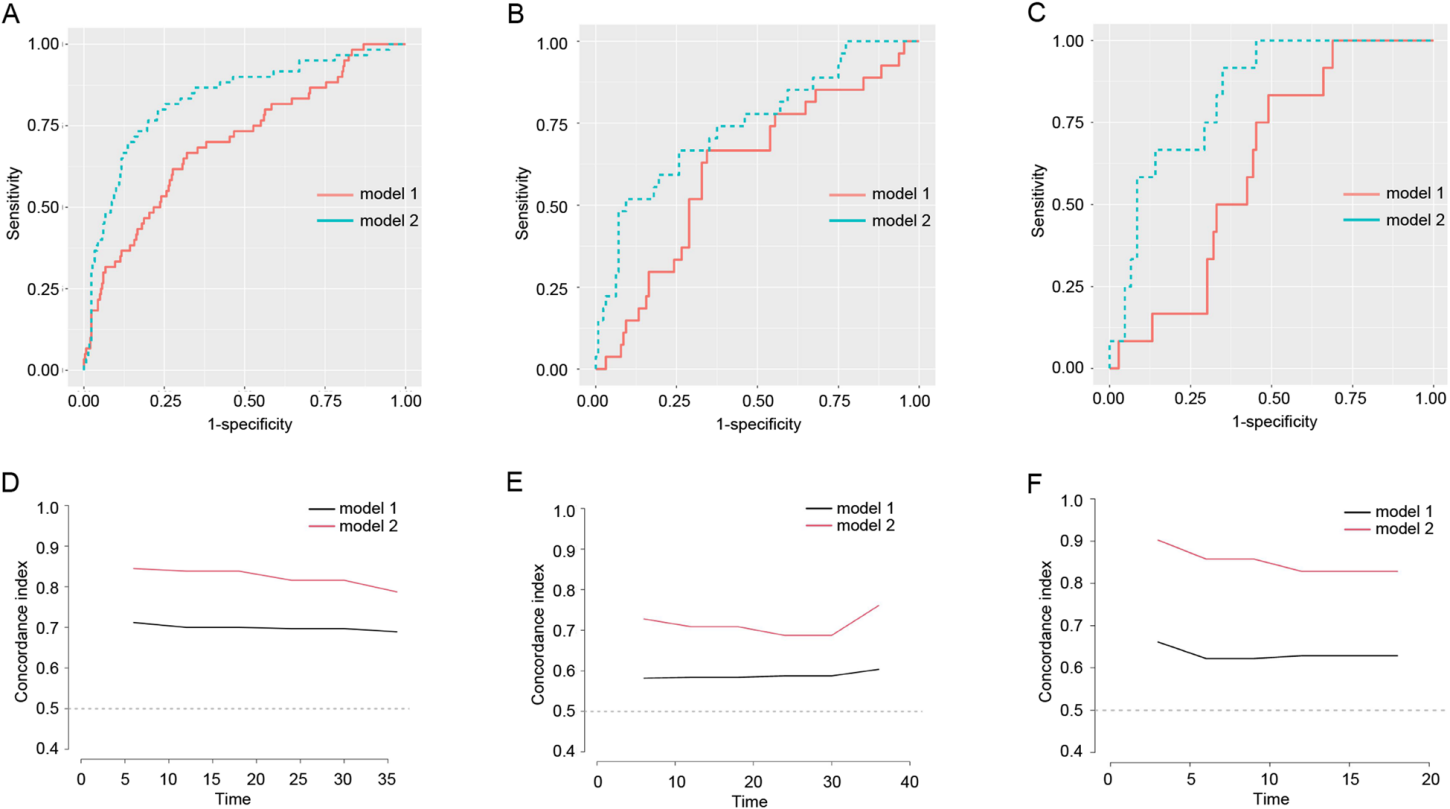
The AUCs and the quantization diagram of C-index and time for two models in the training set, internal test set and external validation set. (**A**) The AUCs of model 1 and model 2 were 0.6988 (95%CI 0.6233–0.7743) and 0.8314 (95%CI 0.7700–0.8927) in training set respectively. The contrast of AUCs: model 1 versus model 2 p < 0.0001. (**B**) The AUCs of model 1 and model 2 were 0.6126 (95%CI 0.5040–0.7291) and 0.7465 (95%CI 0.6387–0.8543) in internal test set respectively. The contrast of AUCs: model 1 versus model 2 *p* = 0.0270. (**C**) The AUCs of model 1 and model 2 were 0.6187 (95%CI 0.5101–0.7475) and 0.8349 (95%CI 0.7381–0.9317) in external test set respectively. The contrast of AUCs: model 1 versus model 2 *p* = 0.0240. (**D**) The quantization diagram of C-index and time for two models in the training set. (**E**) The quantization diagram of C-index and time for two models in the internal test set. (**F**) The quantization diagram of C-index and time for two models in the external validation set.

**Figure 4:**
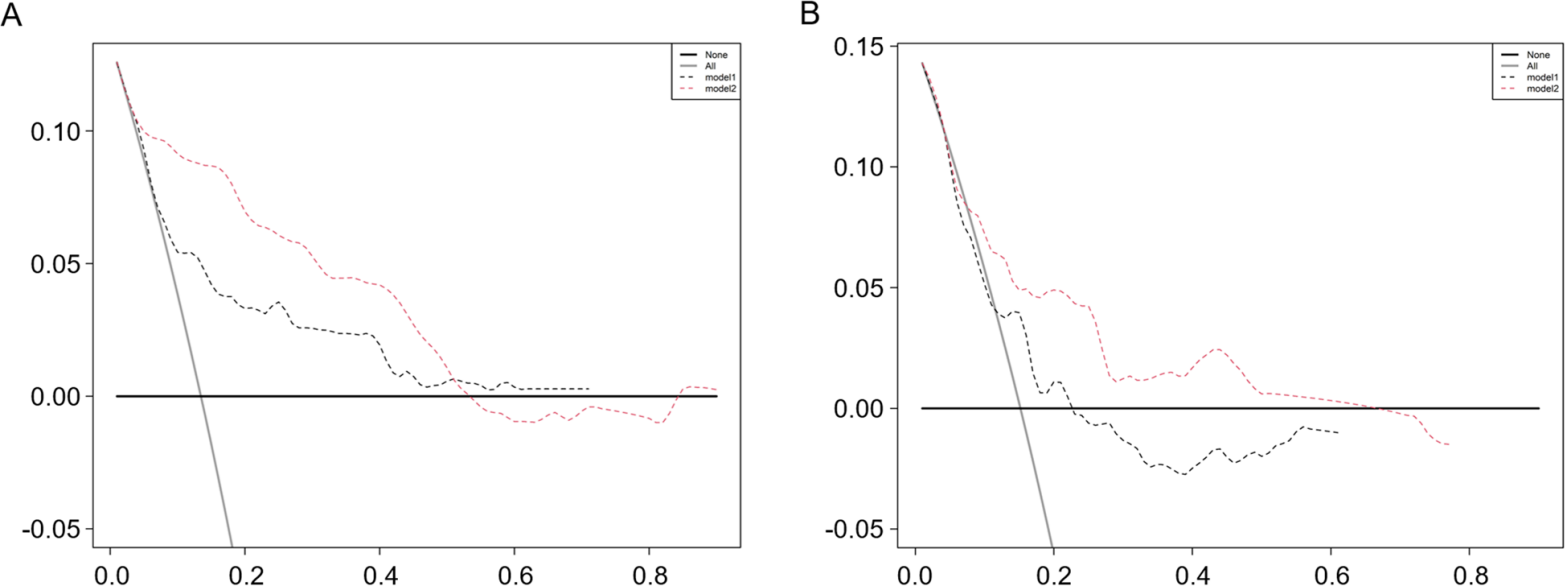
According to the DCA curves of the training set (**A**) and test set (**B**), model 2 was better than the model 1.

Then we established another model (model 2) using recipient’s age, anhepatic phase, eGFR at 30 days after LT and TG levels at 30 days after LT (Fig. 2B). The AUCs of model 2 in training set, internal test set and external validation set were 0.8314 (95%CI 0.7700–0.8927), 0.7465 (95%CI 0.6387–0.8543) and 0.8349 (95%CI 0.7381–0.9317) respectively, showing that model 2 had much better predictive ability than model 1 (Fig. 3). According to the DCA curves of the training set and test set, model 2 was better than model 1 as well (Fig. 4).

### Development and validation of a nomogram model

**Figure 5:**
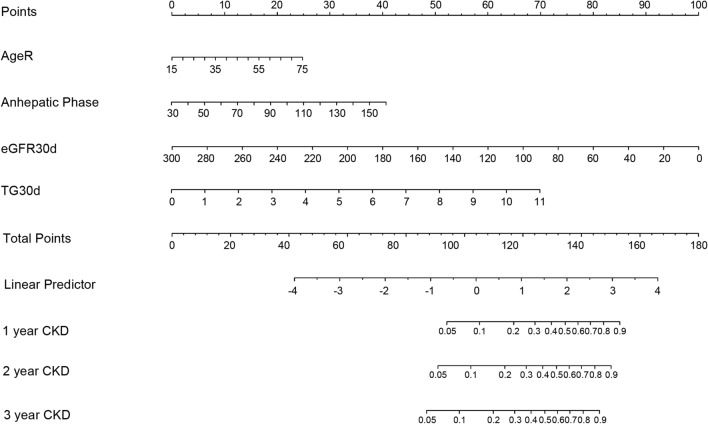
CKD prediction nomogram. Each of these four variables corresponded to a point for LT recipients. The points were added to give a total point that corresponded to the incidence of CKD at different times. Abbreviations: *AgeR* The recipient’s age (year); *Anhepatic Phase* Anhepatic phase (min); *eGFR30d* Glomerular filtration rate at 30 days after LT (mL/min/1.73 m^2^); *TG30d* Triglyceride levels at 30 days after LT (mmol/L).

The four risk factors in model 2 were included to develop a nomogram model to exactly predict the probability of post-LT CKD in each patient at different time point according to the scores (Fig. 5). For example: A 45-year-old liver recipient with anhepatic phase of 80 min and eGFR of 80 ml/min/1.73 m^2^ and TG of 2.0 mmol/L at 30 days after LT. The calculated score was 114 and the probabilities of this recipient developing CKD at 1-, 2- and 3-year after LT were 18%, 20% and 25% respectively.

**Figure 6:**
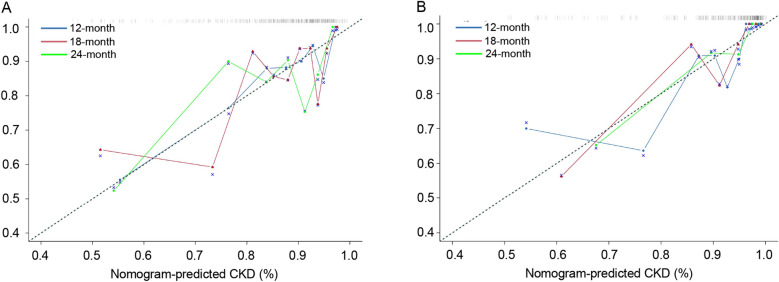
The calibration curves for CKD prediction in the nomogram were consistent with the actual observation in internal test set (**A**) and external validation set (**B**).

The calibration curve of the nomogram was presented in Fig. 6, demonstrating that the post-LT CKD probabilities predicted by the nomogram was consistent with the actual observation in the internal test set and external validation set. It indicated that the nomogram model could accurately predict the risk of post-LT CKD.

## Discussion

CKD is a common long-term complication after LT. Previous studies have revealed that the incidence of post-LT CKD varied widely^2–10^, which mainly due to the different definition of post-LT CKD and the duration of follow-up time. In a single-center retrospective study, Schmitz et al.^3^ showed that 11.7% of liver recipients developed CKD, which was defined as serum creatinine ≥ 1.8 mg/dL ≥ 2 weeks, within 12 months. When the follow-up reached 53.2 months, Fabrizi et al.^8^ demonstrated that 28% of liver recipients developed CKD, based on the definition of eGFR < 60 mL/min/1.73 m^2^ ≥ 3 months. Using a strict criteria according to KDIGO 2012, we showed the incidence of post-LT CKD was 16.9% during a median follow-up of 22.73 months, which was consistent with the previous studies.

The relationship between post-LT CKD and adverse prognosis is still a matter of substantial debate. It has been reported that the occurrence of CKD is not associated with a worse survival^11,22^. In this study, we also found that post-LT CKD did not reduce the patient survival. However, both our and previous studies showed that as the development of CKD, a severe type (GFR < 30 mL/min/1.73 m^2^ or those need renal replacement therapy) was significantly associated with higher mortality after LT^2,23^. LaMattina et al.^10^ retrospectively analyzed 1151 adult deceased LTs and revealed that 3%, 7% and 18% of recipients developed ESRD at 5, 10 and 20 years, which suggested increased incidence of severe CKD with prolonged follow-up time. Thus, there is greatly necessary to identify earlier CKD and cooperate with nephrologists to take early intervention.

In this study, we established a novel nomogram model to accurately predict post-LT CKD. We found it was difficult to predict the occurrence of post-LT CKD before LT because the AUC of model 1 with preoperative parameters was relatively low. In contrast, when we integrated the postoperative parameters, the predictive ability of model was dramatically improved. The independent risk factors for post-LT CKD were recipient’s old age, prolonged anhepatic phase, low eGFR at 30 days after LT and high TG levels at 30 days after LT.

Advanced age is a well-recognized risk factor for progressive kidney dysfunction after LT^2,9^. Here, we found that for each 1 year increased of age, the risk of CKD increased by 3%, which confirmed the above association. Older liver recipients are commonly at higher risk of preexisting kidney dysfunction, metabolic disorders, hypertension, diabetes, cardiovascular diseases, making it susceptible to CKD after LT^2,24,25^. Given that the declining clearance of immunosuppressive agents in the elderly, they are more likely to be affected by immunosuppressor nephrotoxicity, thereby accelerating the development of post-LT CKD^24,26^.

During LT, the inferior vena cava was partially blocked while the portal vein and hepatic artery were completely occluded, strongly impacting the hemodynamics changes in recipients. Additionally, decreased GFR and increased markers of renal injury (e.g., β2-microglobulin, N-acetyl-β-D-glucosaminidase, syndecan-1) were observed during the anhepatic phase, potentially resulting in post-LT CKD^27,28^. In addition, prolonged anhepatic time could increase the risk of graft dysfunction and metabolic disorders, mainly due to the accumulation of cytokines (e.g., interleukin 6), metabolites and other toxicants, and finally deteriorated renal function and reduced patient survival^29^.

Previous study has showed that high levels of TG were significantly associated with low eGFR in liver recipients^30^. In our present study, we also found that TG level at 30 days after LT was an independent risk factor for post-LT CKD, suggesting the potential role of hyperlipidemia in CKD development. In patients with CKD, the altered lipid profile includes elevated low-density lipoprotein cholesterol and very low-density lipoprotein cholesterol and reduced high-density lipoprotein cholesterol^31–33^. Such alteration could result in increased lipid content (e.g., total cholesterol, TG), which accumulates in kidney and triggers lipid nephrotoxicity^34^. Given that the close correlation between hyperlipidemia and CKD, it is necessary to regulate lipid metabolism in recipients at higher risk of post-LT CKD^35^. A series of clinical trials have indicated the use of statins with or without ezetimibe could prevent and treat CKD^36–41^. Moreover, our previous studies also demonstrated that well controlled glucose and lipid levels during the perioperative period of LT could reduce the incidence of post-LT chronic diseases such as metabolic disorders and CKD^42–45^.eGFR is commonly served as an indicator of CKD due to its detection is accurate and convenient. In our study, we indicated that low eGFR level at 30 days after LT was an independent risk factor of post-LT CKD, suggesting the renal dysfunction in the early post-LT period may contribute to the development of CKD. In a retrospective cohort study, Sato et al.^46^ revealed that eGFR < 60 mL/min/1.73 m^2^ at 1 month following LT was a predictive factor of CKD at 2-year after LT, which proved our hypothesis. During organ procurement and implantation, blood loss could result in intraoperative or post-LT renal injury, thereby influencing the eGFR^46^. These results highlight the importance of controlling the early post-transplant eGFR. In addition, efforts should be made to avoid massive intraoperative blood loss during LT, usage of nephrotoxic medications and acute kidney injury.

There are several limitations in our study. Firstly, it was an observational study with relatively limited cases and short follow-up time. CKD is a slowly progressive disease with increased incidence as the follow-up time prolonged. Secondly, the nomogram model needs to be validated in prospective studies with well design. Thirdly, given that the fewer ESRD cases in our study, large-scale and adequately powered studies with more samples are necessary to determine independent risk factors of ESRD and construct believable models to identify who deteriorate to ESRD and who doesn’t. At last, although CLTR included more than twenty thousand cases of LTs during 2017–2020, only 118 had sufficient follow-up data to define post-LT CKD. The follow-up data input and management of the national database are needed.

## Conclusion

As a long-term complication after LT, severe post-LT CKD could result in a significantly reduced survival rate in liver recipients. Prompt management of dyslipidemia and renal dysfunction during the early post-LT period may of help to prevent the development of post-LT CKD. Furthermore, we established a novel nomogram to predict post-LT CKD, showing an excellent diagnostic efficacy.

## Data Availability

The data that support the findings of this study are available from China Liver Transplant Registry (CLTR) but restrictions apply to the availability of these data, which were used under license for the current study, and so are not publicly available. Data are however available from the corresponding author upon reasonable request and with permission of CLTR.

## Abbreviations

AUC: The area under the curve
BMI: Body mass index
CLTR: China Liver Transplant Registry
CKD: Chronic kidney disease
DCD: Donation after circulatory death
eGFR: Estimated glomerular filtration rate
LT: Liver transplantation
MDRD: Modified diet in renal disease
TG: Triglyceride
